# Separated Sexes, Shared Consequences: Disentangling Predictors of Genetic Diversity in Dioecious Angiosperms

**DOI:** 10.1002/ece3.73459

**Published:** 2026-05-10

**Authors:** Thais Martins Teixeira, Alison Gonçalves Nazareno

**Affiliations:** ^1^ Department of Genetics, Ecology and Evolution Federal University of Minas Gerais Belo Horizonte Minas Gerais Brazil

**Keywords:** angiosperms, conservation, dioecy, genetic diversity, pollination, sexual system

## Abstract

Sexual systems such as dioecy, although rare in angiosperms, seem to enhance intrapopulation genetic diversity due to obligate outcrossing. Empirical studies had strengthened theoretical predictions that genetic diversity in dioecious angiosperms is strongly linked to population demographic structure, phenological patterns, and species life‐history traits such as pollination mode and seed dispersal syndrome. As anthropogenic pressures and climate change have disrupted and altered plant reproduction, understanding how ecological and environmental factors modulate genetic diversity in threatened dioecious angiosperms is paramount to inform and to establish effective conservation and management plans. Here, we compiled genetic and ecological data of 66 dioecious angiosperms from articles published until August 2025. To test the effects of pollination mode, seed dispersal syndrome, reproductive mode, growth form, conservation status, endemism, and species distribution range on intrapopulation genetic diversity (i.e., *H*_E_), we implemented Generalized Linear Mixed Models fitted with Template Model Builder. Our results revealed that species conservation status and endemism play a significant role in the patterns of *H*_E_ among dioecious angiosperms, with endemic and/or threatened species showing reduced levels of genetic diversity. Pollination mode also emerged as an important predictor of *H*_E_, with abiotically pollinated species exhibiting highest genetic diversity. Pruning not only the knowledge on how ecological and evolutionary processes drive molecular variation in dioecious angiosperms, our study is also an attempt to strengthen conservation efforts to plant species presenting rare sexual system. We stress that dioecious angiosperms should be targeted as priority in conservation agendas worldwide.

## Graphical abstract

This study evaluates the factors influencing intrapopulation genetic diversity in dioecious flowering plants by integrating genetic and ecological data from studies published up to 2025. We show that pollination mode, endemism, and conservation status significantly shape genetic diversity patterns, with endemic and threatened species exhibiting the lowest levels. These findings highlight the need for increased conservation attention toward dioecious species, emphasizing their vulnerability and the importance of integrating genetic metrics into decision‐making frameworks.

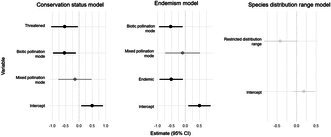

## 1 Introduction

Reproduction is a biological process essential to the persistence and evolutionary success of all living organisms. In flowering plants (i.e., angiosperms), a plethora of sexual (e.g., monoecy, andromonoecy, dioecy, gynodioecy, trimonoecy) and mating systems (i.e., autogamy, geitonogamy, allogamy) can be found, reflecting the remarkable evolutionary flexibility of angiosperms in their reproductive strategies (Barrett 2002). The diversity of sexual reproduction mechanisms has important evolutionary implications as it may ameliorate genetic variation and reduce, or inflate, the levels of inbreeding and genetic differentiation (Bawa and Beach 1981; Barrett 2002; Charlesworth 2006; Ellegren and Galtier 2016; Hamrick and Godt 1996). Nonetheless, the evolutionary advantages and disadvantages of distinct sexual reproduction mechanisms remain a topic of ongoing debate, as each strategy entails specific costs and benefits that may vary depending on ecological and evolutionary contexts (Barrett 2002; Charlesworth 2006; Darwin 1877; Renner 2014).

The costs and rewards of dioecy, for instance, have been widely discussed since it occurs only at 6% of known flowering plants (K. S. Bawa 1980; Renner 2014). Classical evolutionary theories proposed that dioecy evolved primarily as an adaptation to selective pressures to reduce inbreeding and enhance genetic variation, as it requires outcrossing mates (Ainsworth 2000; Darwin 1876). As a matter of fact, empirical studies have suggested that dioecy may contribute to maintaining high levels of genetic diversity, as it may increase the likelihood of crossing between non‐related individuals and attenuate the deleterious effects of inbreeding as a consequence (e.g., Cascante‐Marín et al. 2020; De Oliveira Melo and Franceschinelli 2016; Fuchs et al. 2023; Luna et al. 2007; Mooney et al. 2010; Muyle et al. 2021; Sato et al. 2006). However, this “win” situation seems not to be the rule of thumb to all dioecious plant species. For instance, contrasting levels of genetic diversity have been reported to dioecious plants with distinct range size distribution, pollination mode, seed dispersal syndrome, growth form, and demographic conditions (Cantley et al. 2023; Luna et al. 2007; Hamrick and Godt 1990, 1996; Nybom 2004; De Kort et al. 2021; Hoban et al. 2022). Such findings suggested that ecological and environmental factors may modulate the genetic outcomes associated with dioecy.

Life‐history traits directly linked to gene flow, such as pollination mode and seed dispersal syndrome, may be particularly relevant, as they influence both the magnitude and spatial scale of pollen and seed movement within and among populations (Aguilar et al. 2019; Browne et al. 2018; Gamba and Muchhala 2020). Specifically in dioecious plants, some empirical evidence suggests that abiotic pollination and biotic seed dispersal may be associated with higher levels of intrapopulation genetic diversity (Llorens et al. 2017; Paschoa et al. 2018; de Jesus Aguilar‐Aguilar et al. 2023). Although mating systems are often considered the primary explanation for high levels of genetic variation (Paschoa et al. 2018), the role of pollination mode and seed dispersal syndromes in modulating genetic patterns in this distinct group of plants remains poorly understood. Indeed, some intrinsic features of dioecious systems may counteract such expected genetic advantages of obligate outcrossing. One potential constraint is the seed‐shadow handicap, which arises because only female individuals produce seeds in dioecious populations (Baker 1955; Lloyd and Bawa 1984; Heilbuth et al. 2001). As a consequence, the spatial distribution of seeds may be reduced compared with cosexual populations in which all individuals contribute to propagule production, increasing the likelihood of local clustering of related offspring and competition among siblings, particularly under limited seed dispersal (Lloyd and Bawa 1984; Heilbuth et al. 2001). An analogous process may also occur via pollen dispersal, albeit typically to a lesser extent, given that pollen is generally dispersed over longer distances than seeds (Lloyd and Bawa 1984; Ohya et al. 2017). In this context, the effectiveness of both seed dispersal and pollen flow becomes critical, as theoretical models suggest that dioecy evolves and persists more readily in species with wide dispersal extent (Lloyd and Bawa 1984; Fromhage and Kokko 2010).

Growth form (i.e., habit) has also been recognized as a relevant predictor of genetic diversity in plants, with previous studies reporting higher levels of genetic variation in trees compared to herbaceous species (e.g., Carvalho et al. 2019; Chung et al. 2020; Hamrick et al. 1992), an expected pattern due to differences on lifespan (Chung et al. 2020; Olson et al. 2016). Because dioecious species are obligatorily outcrossing, it seems to be pertinent to investigate whether habit affects the amount of genetic diversity of dioecious flowering plants. In addition, reproductive mode may further shape the genetic patterns in dioecious flowering plants: while sexual reproduction in dioecious plant species may enhance genetic variation through recombination and mutation, asexual reproduction seems to reduce the levels of genetic diversity and effective population sizes (Waycott et al. 1996) due to a biased morph ratio in populations with extensive clonal growth or vegetative dispersal (Barrett 2015).

Beyond life‐history traits, geographic and conservation related factors are also expected to influence genetic diversity. Endemism (i.e., habitat specificity; Kruckeberg and Rabinowitz 1985) often implies habitat specialization and geographic isolation, potentially limiting gene flow and increasing susceptibility to genetic erosion (Babbel and Selander 1974; Liveri et al. 2024). Similarly, populations of species with narrow distribution ranges often exhibit reduced genetic variation than widespread ones (e.g., Diaz et al. 2012; Ellstrand and Elam 1993; Gitzendanner and Soltis 2000; Gibson et al. 2008; Karron 1987; Maebe et al. 2016).

As species conservation status is mirrored by the intensity of population decline (Edgar 2025; IUCN 2018; López‐Pujol et al. 2009; Schmidt et al. 2023; Spielman et al. 2004), it can also be a relevant predictor of intrapopulation genetic diversity. Species conservation categorization is based on criteria that include population size, rate of decline, area of geographic distribution, and the degree of fragmentation or restriction of habitats (IUCN 2018). Therefore, species that fall into threatened categories, according to the population genetic theory of small populations, are more likely to experience genetic erosion due to reduced population size, restricted gene flow, and inbreeding, all of which can reduce genetic variation within populations, increasing extinction risk (e.g., Ellstrand and Elam 1993; Forester et al. 2022; Frankham 1995; Gilpin and Soulé 1986; Hamrick and Godt 1990; Primack and Rodrigues 2001; Spielman et al. 2004; Teixeira and Nazareno 2021). Indeed, while dioecy may promote genetic diversity under stable demographic conditions (i.e., populations without unbiased sexual ratio), it may erode genetic variation in species experiencing ongoing population decline due to anthropogenic economic activities (Broadhurst 2011; Dubreuil et al. 2010; Lauterbach et al. 2012; Sherman‐Broyles et al. 1992; Vandepitte et al. 2009). A comprehensive survey identified that dioecious clades have, proportionally, more species listed as threatened with extinction than other plant groups (Vamosi and Vamosi 2005).

Whilst there is support to the idea that dioecious species are more prone to extinction than those with other sexual systems (Barrett 2002; Heilbuth 2000; Vamosi and Vamosi 2005), a systematic review of how ecological and environmental factors operate on genetic diversity in dioecious flowering plants is required to inform and guide effective conservation and management actions (Broadhurst et al. 2017; López‐Pujol et al. 2009). Therefore, we compiled genetic and ecological data of dioecious flowering plants from articles published until August 2025. To test the effects of pollination mode, seed dispersal syndrome, reproductive mode, growth form, conservation status, endemism, and species distribution range on intrapopulation genetic diversity, we implemented Generalized Linear Mixed Models fitted with Template Model Builder. These models allowed the incorporation of both fixed and random effects, providing a comprehensive and robust analysis of how the tested variables shape the genetic variation across dioecious flowering plants. Pruning not only the knowledge on how ecological and evolutionary processes drive genetic variation in dioecious flowering plants, our study is an attempt to strengthen conservation efforts to this unique set of plants. We emphasize that threatened dioecious flowering plants should be targeted as a priority in conservation agendas worldwide.

## 2 Methods

### 2.1 Literature Surveyed

**FIGURE 1:**
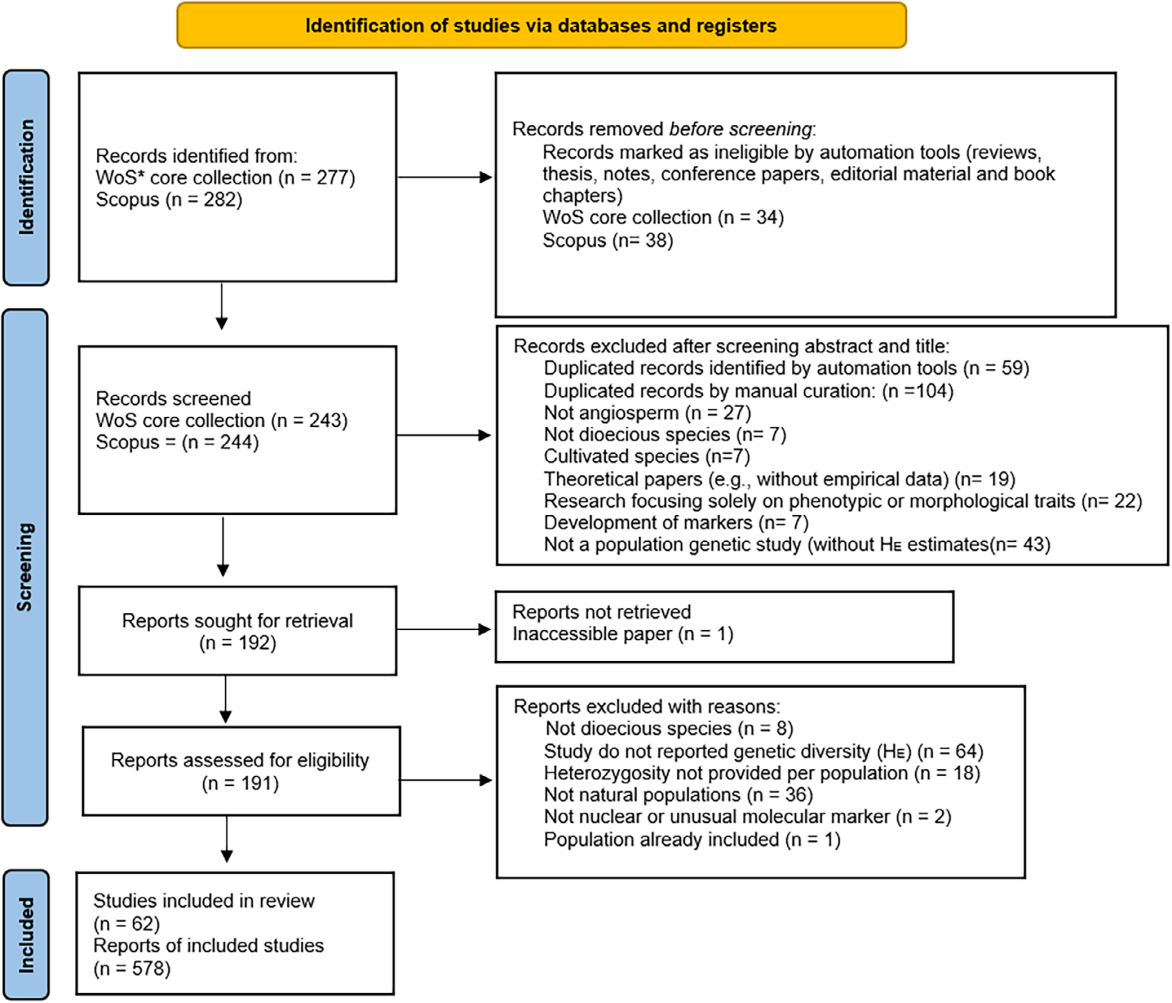
PRISMA (Preferred Reporting Items for Systematic Reviews and Meta‐Analyses) flow chart detailing the steps followed during articles collection. WoS, Web of Science.

To investigate the general patterns of intrapopulation genetic diversity in dioecious flowering plants, we conducted a comprehensive search on Scopus and Web of Science employing the strings ((“genetic diversity” OR “population genetic*” OR “conservation genetic*” OR “population genomic*” OR “conservation genomic*”) AND (“dioec*”) AND (“plant*”) NOT (“gymnosperm*”)) targeting research articles that reported genetic diversity (i.e., *H*_E_) for dioecious flowering plants published from inception until August 2025. After removing duplicates, we screened titles, keywords, and abstracts of the retrieved manuscripts and excluded those that did not meet the inclusion criteria (Figure 1). Articles deemed potentially relevant were retained for further full‐text review, following the latest PRISMA statement (Page et al. 2021).

In the dataset, each observation corresponds to one local population, defined as a group of individuals of the same species, in a specific geographic area as defined by authors. For each observation, we extracted the following information: botanical nomenclature; estimates of expected heterozygosity (*H*_E_), life‐history traits (e.g., pollination mode, seed dispersal syndrome, growth form, and reproductive mode), endemism, species distribution range, and conservation status. We also carefully reviewed and updated botanical nomenclature for each observation based on databases such as Global Biodiversity Information Facility (GBIF, https://www.gbif.org) and Plants of the World Online (https://powo.science.kew.org), for details see Table S1.

The predictor variables were encoded as categorical factors with two to three levels to reduce model complexity due to within‐category sample sizes (see Table 1). Briefly, we reclassified as biotic (i.e., animal‐mediated), abiotic (i.e., wind‐, water‐, and/or gravity‐mediated), or mixed, when more than one mode was reported. Regarding the growth form, each species was categorized as tree, shrub or non‐woody, that includes herbaceous species, epiphytes and hemiepiphytes, and geophytes. Reproductive mode was classified as dioecious sexual or dioecious with asexual reproduction (e.g., vegetative growth or apomixis). Species distribution range was assigned based on information reported in the original studies and reflects the extent of species' occurrence (restricted vs. widespread). Endemism was defined broadly and coded as a binary variable, with species reported as endemic at any spatial scale (e.g., local, country, biome/ecosystem, or habitat) classified as endemic, and all remaining species classified as non‐endemic. Conservation status was coded as threatened for species classified as Vulnerable (VU), Endangered (EN), or Critically Endangered (CR), and as not threatened for those listed as Near Threatened (NT), Least Concern (LC), or Data Deficient (DD), following IUCN or other red lists. We also included three factors that may affect *H*_E_ estimate: type and inheritance of molecular marker, number of loci, and sample size or number of multiple locus genotyping for clonal species.

**TABLE 1:** Summary of the number of representatives based on literature survey on each class from the variables tested as predictors of population genetic diversity on dioecious flowering plants included as fixed effects in the Generalized Linear Mixed Models.

Category	Species	Populations
**Seed dispersal syndrome**		
Abiotic	21	159
Abiotic and biotic	7	57
Biotic	37	360
Unknown	1	2
**Species distribution range**		
Widespread	46	464
Restricted	20	114
**Endemism**		
Not endemic	47	473
Endemic	19	105
**Growth form**		
Tree	29	254
Non woody^a^	16	96
Shrub	21	228
**Pollination mode**		
Abiotic	18	143
Abiotic and biotic	8	54
Biotic	39	379
Unknown	1	2
**Reproductive mode**		
Asexual dioecious^b^	31	270
Dioecious	35	308
**Conservation status**		
Not threatened^c^	53	510
Threatened^d^	13	68

^a^ Include herbaceous, epiphytes, and hemi‐epiphytes species.

^b^ Include species reproducing by apomixis.

^c^ IUCN conservation categories: Vulnerable (VU), Endangered (EN), and Critically Endangered (CR).

^d^ Species classified in one of the not endangered IUCN conservation categories (non‐endangered): Near Threatened (NT), Least Concern (LC), and data deficient (DD).

Overall, we kept in the dataset only studies that provided measures of *H*_E_ averaged across loci obtained from adult plants sampled in situ. For species with distinct sexual morphs (i.e., dioecious, monoecious and/or hermaphroditic), we used only data from entire dioecious populations. To fill the lack of information related to species life‐history traits and conservation status, we researched those on related literature and distinct databases (e.g., Plant Trait Database, https://www.try‐db.org; Kattge et al. 2011; The IUCN Red List of Threatened Species, https://www.iucnredlist.org).

### 2.2 Response Variable

To investigate the predictors of the genetic diversity among dioecious flowering plants, we used the expected heterozygosity (Nei 1973) as response variable, as it seems to be less sensitive to sampling efforts than other estimates (Eugenia Barrandeguy and Victoria García 2021; Kanaka et al. 2023; Nazareno and Jump 2012; Toro et al. 2009). Taking into account that distinct molecular markers (e.g., allozymes, SSR, AFLP, SNP, RAPD, and ISSR) were used among studies, and that *H*_E_ is affected by molecular marker inheritance, reaching up 0.5 (biallelic markers) to 1.0 (multiallelic markers), we standardized and normalized *H*_E_ estimates to range from nil to one as proposed by De Kort et al. (2021).

### 2.3 Prior Analyses

Because species life‐history traits are often evolutionarily and/or ecologically associated, predictor variables may not vary independently. For example, conservation status is intrinsically linked to species distribution patterns, as taxa with narrow geographic ranges or restricted habitat requirements (i.e., endemics) tend to be more prone to extinction than widespread species (Leão et al. 2014; Purvis et al. 2000; Staude et al. 2020). Similarly, growth form has been identified as a potential correlate of plant extinction risk, with epiphytes being more prone to extinction than other life forms (Leão et al. 2014; Sodhi et al. 2008; Turner et al. 1994). Moreover, dioecy is frequently associated with particular reproductive traits, such as wind pollination and animal‐mediated seed dispersal (Bullock 1994; Renner 2014; Vamosi et al. 2003). Consequently, some combinations of predictor variables may occur more often than expected by chance (Draper et al. 2023), potentially leading to correlations among predictors and violating the assumption of independence required for reliable model inference.

Considering these potential associations, we evaluated possible dependencies among predictor variables in preliminary analyses prior to model fitting. First, we performed Fisher's exact tests of independence among all pairs of predictor variables using the R function fisher.test. The strength of the relationship was estimated using Cramer's *V* (Cramér 1946). We considered moderate to low association values < 0.3.

We also quantified the phylogenetic signal, which corresponds to the statistical dependence among species trait values due to their phylogenetic relatedness (Revell et al. 2008). The absence of independence can lead to inflated Type I error rates when assessing relationships between traits (Revell 2010). To investigate variable sensitivity to phylogenetic signal, we used the most recent version of the R package U.PhyloMaker (Jin and Qian 2019, 2022) to construct a species‐level phylogeny based on the megatree GBOTB.extended.WCVP.tree (Jin and Qian 2022; Smith and Brown 2018; Zanne et al. 2013). Based on the resulting tree, we used the R package phytools (Revell 2012) to estimate Pagel's *λ* (Pagel 1999), testing whether the response variable fits the assumption of phylogenetic signal independence. For categorical variables, we used Abouheif's *C*_mean_ method implemented with the function abouheif.moran (method = “oriAbouheif”) in the R package adephylo (Abouheif 1999; Jombart et al. 2010). The significance of both tests considered 1000 simulations.

### 2.4 Generalized Linear Mixed Models

**FIGURE 2:**
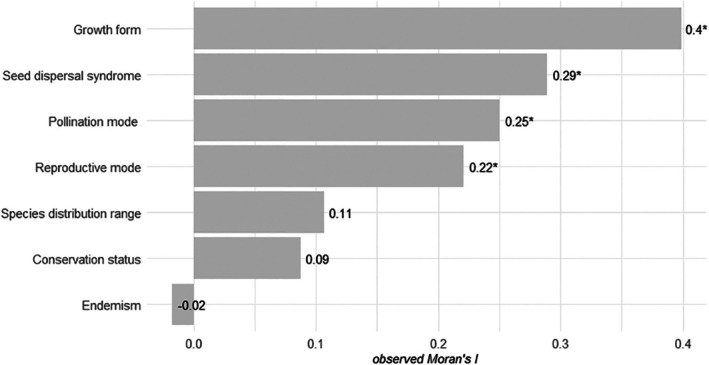
Phylogenetic signal across ecological and life‐history traits estimated using Abouheif's test (abouheif.moran, method = OriAbouheif). Moran's *I* is a statistic based on spatial autocorrelation, adapted for phylogenetic data to quantify the tendency of closely related species to share the similar trait values. The coefficient ranges from −1, indicating negative correlation, to +1, indicating strong positive correlation, while 0 denotes phylogenetic independence. Growth form exhibited the highest phylogenetic signal, followed by seed dispersal syndrome, pollination mode, and reproductive mode. Despite the significant correlations (*p* < 0.05), the observed Moran's *I* suggests moderate to low signal strength.

Despite some life‐history traits (growth form, *I* = 0.40; reproductive mode, *I* = 0.2; pollination mode *I =* 0.25; and seed dispersal syndrome, *I* = 0.29) exhibited a significant phylogenetic signal (*p* < 0.05; Figure 2), the normalized response variable was phylogenetically independent (*λ* = 0.435, LR = 1.55, *p* = 0.214). Therefore, to assess the relative contribution of selected predictors to intrapopulation genetic diversity in dioecious flowering plants, we implemented Generalized Linear Mixed Models using the glmmTMB package in R (Brooks et al. 2017).

Considering that the response variable represents continuous data bounded between zero and the unit, and that it does not fit a Gaussian distribution, we performed the models using the beta distribution (McDonald and Xu 1995) with a logit link function to take a linear combination of the covariate values (MacKenzie et al. 2018). To eliminate extreme values from our dataset, the response variable was slightly adjusted to remain strictly within the open interval between zero and the unit. To account for the high associations among conservation status, endemism, and species distribution range (Φc > 0.49, *p* < 0.01; see more details in the Results and on Table 2), we constructed three separate comprehensive models, each incorporating one of these variables as a predictor. In all models, we included pollination mode, seed dispersal syndrome, reproductive mode, and growth form as fixed effects. Species and molecular markers were included as random effects to account for phylogenetic and potential methodological biases. As population genetic estimates robustness is sampling design dependent (De Kort et al. 2021; Nazareno and Jump 2012; Nazareno et al. 2017), and considering that sampling scheme (e.g., number of individuals/populations sampled, and molecular marker's type, inheritance mode and genome representativity [i.e., sample efforts]) varied among studies, models residuals were weighted employing the function $\omega =\frac{k}{\sum_{n=0}^{n=k}\frac{1}{k}\log n}$ (*k* and *n* represent the number of weighted variables; see Nakagawa and Cuthill 2007).

**TABLE 2:** Independence test of categorical variables—Fisher test for Count Data with simulated *p*‐value (based on 2000 replicates). In bold, combinations with significant *p*‐value and Cramer *V* (Φc) higher than 0.3, which indicates moderate to high associations.

Paired variables	*p*	Φc
Conservation status—Species distribution range	**0.000**	**0.668**
Conservation status—Endemism	**0.000**	**0.527**
Conservation status—Pollination mode	0.668	0.149
Conservation status—Seed dispersal syndrome	0.148	0.256
Conservation status—Reproductive mode	0.549	0.084
Conservation status—Growth form	0.266	0.204
Species distribution range—Endemism	**0.000**	**0.673**
Species distribution range—Pollination mode —	0.277	0.258
Species distribution range—Seed dispersal syndrome	**0.043**	**0.351**
Species distribution range—Reproductive mode	1.000	0.026
Species distribution range—Growth form	0.282	0.200
Endemism—Pollination mode —	0.274	0.243
Endemism—Seed dispersal syndrome	0.064	0.334
Endemism—Reproductive mode	0.055	0.263
Endemism—Growth form	0.320	0.187
Pollination mode—Seed dispersal syndrome	0.099	0.435
Pollination mode—Reproductive mode	0.906	0.137
Pollination mode—Growth form	0.234	0.256
Seed dispersal syndrome—Reproductive mode	0.612	0.193
Seed dispersal syndrome—Growth form	**0.004**	**0.377**
Reproductive mode—Growth form	0.151	0.239

The effect of the different combinations of each variable in the three models was assessed using the “dredge” function in the R package MuMln (Bartoń 2010). Instead of only considering the variables included in models with a delta Akaike information criteria (ΔAICc) equal to zero, we calculated the relative importance (RI) of all predictors using the method proposed by Burnham and Anderson (2004). The RI of each predictor was calculated as the sum of the Akaike weights of all models in which the predictor was included, considering only models with ΔAICc < 4 (Burnham and Anderson 2004; De Kort et al. 2021). To convert RI as a percentage, each value was divided by the total sum of Akaike weights from all models within the ΔAICc < 4 threshold. As a result, predictors with RI > 50% were retained as fixed effects in the three final models to estimate the variable parameters. Predictor significance of the best supported models was assessed through Analysis of Variance (ANOVA) from the R package car (Fox and Weisberg 2019).

## 3 Results

### 3.1 Literature Surveyed

Following the selection of studies that filled the inclusion criteria, a total of 191 articles quantifying intrapopulation genetic diversity in natural populations of dioecious flowering plants were assessed for eligibility (see Supporting Information). After screening and full‐text review procedures, a total of 62 articles encompassing 66 taxa (43 genera, 32 families, and 19 orders) were kept in the dataset. Based on the most recent estimate of the frequency of dioecy in angiosperms, our dataset represents approximately 18% of the families reported by Renner (2014). The sampled taxa were taxonomically diverse, although some families were more frequently represented, particularly Lauraceae (*n* = 7), Anacardiaceae, Arecaceae, Moraceae, and Salicaceae (*n* = 5 each). At the genus level, *Lindera* (Lauraceae), *Chamaedorea* (Arecaceae), *Salix* (Salicaceae), and *Rhus* (Anacardiaceae) accounted for multiple taxa in the dataset (see Supporting Information). The majority of these studies (*n* = 32) used microsatellite markers to estimate *H*_E_ (Figures S1 and S2). Information related to sample sizes computed for each predictor are summarized in Table 1. Briefly, most species in our dataset presented biotic seed dispersal (*n* = 37), followed by abiotic (*n* = 21) and mixed dispersal (*n* = 7), while one species had an unknown dispersal syndrome. Similarly, biotic pollination predominated (*n* = 39 species), followed by abiotic (*n* = 18) and mixed pollination (*n* = 8), with one species classified as unknown. Most taxa were widespread (*n* = 46) and non‐endemic (*n* = 47 species), whereas 20 species had restricted distributions and 19 were endemic. Trees were the most common growth form (*n* = 29 species), followed by shrubs (*n* = 21) and non‐woody species (*n* = 16). Regarding reproductive mode, 35 species were classified as dioecious and 31 as asexual dioecious. Finally, most taxa were categorized as not threatened (*n* = 53), whereas 13 were classified as threatened.

### 3.2 Prior Analyses

The association tests showed significant *p*‐values (< 0.05) accompanying Cramer *V* values higher than 0.30 for five combinations of categorical traits, which indicates moderate to high associations (Table 2). The strongest association was observed among the three combinations of species distribution range, endemism, and conservation status. In our dataset, more than 79% of the species at an endangered risk class are restricted, and 75% of all restricted species are also endemic. Regarding the direct association between conservation status and endemism, only 23% of the threatened species are not endemic.

In the analyzed dataset, seed dispersal syndrome was significantly associated with both species distribution range and growth form, with a slight tendency of widespread species to exhibit biotic dispersal (*p* = 0.043; Φc = 0.351; Table 2). Additionally, biotic seed dispersal was more common in shrubs and trees than in non‐woody species (*p* = 0.004; Φc = 0.377; Table 2).

**FIGURE 3:**
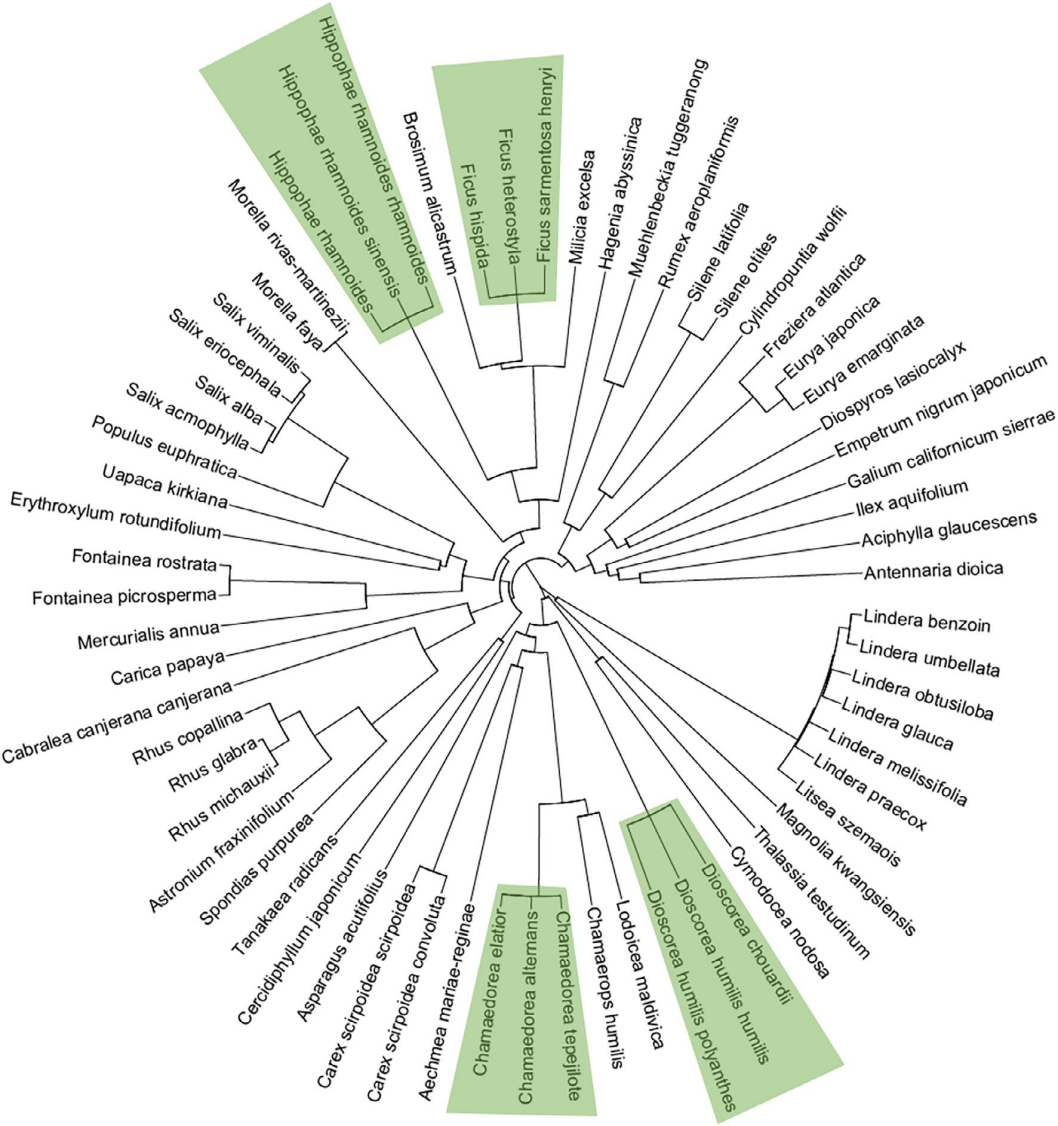
Unrooted phylogenetic tree including the 66 taxa assessed in the quantitative analysis. The integration of subspecies as tips resulted in polytomies for the genus *Dioscorea*, *Hippophae*, and *Ficus*. The polytomy in the *Chamaedorea* branch can be attributed to the unresolved phylogenetic structure within the genus, despite previous studies using both molecular and morphological data.

Taking into account the phylogenetic analysis, 95% of the interspecific relationships in the resulting tree were resolved but some polytomies were observed in four genera (Figure 3). One of them corresponds to three *Chamaedorea* species, possibly indicating that phylogenetic relationships within the genus remain ambiguous despite previous molecular and morphological studies (Cuenca et al. 2009; Thomas et al. 2006). Polytomies were also observed in subspecies of *Dioscorea*, *Ficus*, and *Hippophae*, reflecting a limitation of the backbone tree, not including specific data on subspecies. Nevertheless, the methods we used to detect phylogenetic signal are not sensitive to polytomies (Münkemüller et al. 2012).

### 3.3 Generalized Linear Mixed Models

The results obtained using the “dredge” function revealed the three models (i.e., Endemism, Conservation Status and Species Distribution Range) with the lowest AICc values when different predictors were combined (Table S2). The Conservation status model (AICc = −313.42, Table S2) derived from the combination between “conservation status” and “pollination mode”. The Endemism Model with the lowest AICc (= −314.54, Table S2) included “endemism” and “pollination mode” as fixed effects. For the Species distribution range model, “species distribution range” and “seed dispersal syndrome” were included as predictors (AICc = −312.96, Table S2).

The relative importance (RI) of predictors was calculated for each of the three comprehensive models tested, where one of the three associated predictors (i.e., conservation status, endemism, or species distribution range) was included as a fixed effect along with growth form, seed dispersal syndrome, and pollination and reproductive modes (Figure S3). Focal predictors (i.e., conservation status, endemism, species distribution range) were the most influential variables across all models (Figure S3), with RI values varying from 82.25% (species distribution range) to 93.63% (endemism). Pollination mode consistently emerged as the second most important predictor in all models (Figure S3), but it was only retained in the Conservation status and Endemism models as RI was less than 50% for the Species Distribution Range model (Figure S3).

Random effects accounted for non‐negligible variation in mean expected heterozygosity, with greater variability attributed to species identity than to molecular marker type. The dispersal parameter (*φ* = 26.6) indicated relatively low dispersion around the model‐predicted means. None of the models showed evidence of overdispersion. The distributions of residuals for all final models are presented in Figure S4.

**FIGURE 4:**
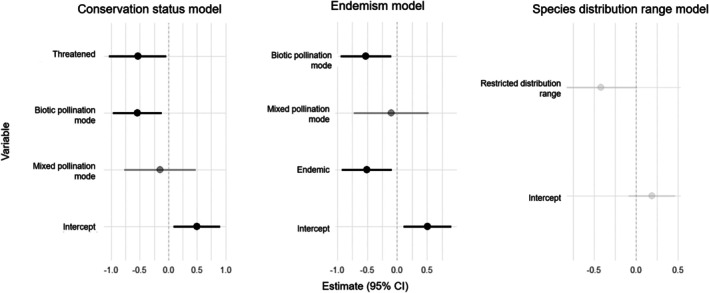
Predictor variables estimates for each model with 95% confidence intervals. Significant effects (i.e., *p* < 0.05) are shown in black. In addition to biotic pollination, threatened status and endemism were considered significant predictors of reduced expected heterozygosity in dioecious flowering plants in Conservation status and Endemism models, respectively. Species distribution range predictor was not statistically significant (*p* = 0.0563).

By investigating the potential predictors of intrapopulation genetic diversity in dioecious flowering plants, we found that the pollination mode was a significant driver of population genetic diversity, with biotically pollinated plants showing lower intrapopulation genetic diversity than abiotically ones (Table 3), a pattern consistently observed across two of the three models. In the ANOVA test, pollination mode was a significant factor in the two final tested models in which it was included (*p*_conservation status_ = 0.03203; *p*_endemism_ = 0.03497; Table 4). Conservation status and endemism predictors were also significant drivers of intrapopulation genetic diversity on Conservation status and Endemism models (Tables 3 and 4, Figure 4). Notably, reduced genetic diversity was observed in endemic species, compared with non‐endemic ones, and in threatened species compared with those not classified as one of the IUCN categories (Table 3, Figure 4). Despite populations of narrow species showing a tendency for lower intrapopulation genetic diversity when compared with widespread species (Table 4, Figure 4), this result was not statistically significant in the Species distribution range model (Tables 3 and 4). The ANOVA test for Species distribution range was congruent with the results of its model (*p* = 0.05632; Table 4).

**TABLE 3:** Summary of the final models, including only predictors with Relative Importance (RI) > 50%. The variables Conservation status (A), Endemism (B), and Species distribution range (C) were treated separately due to high association (Fisher's exact test, *p* < 0.05, Φc > 0.30). Predictor reference levels are indicated in italics. Significant *p*‐values and their corresponding details are in bold.

	Estimate	SE	*z* value	Pr(>|*z*|)
(A) Conservation status				
(Intercept)	0.4966	0.2083	2.384	0.0171
Conservation status				
*Not threatened*				
Threatened	**−0.5342**	**0.2558**	**−2.088**	**0.0368**
Pollination mode				
*Abiotic*				
Biotic	**−0.5433**	**0.2176**	**−2.497**	**0.0125**
Mixed (biotic and abiotic)	−0.1471	0.3187	−0.462	0.6444
(B) Endemism				
(Intercept)	0.5085	0.2020	2.518	0.0118
Endemism				
*Not endemic*				
Endemic	**−0.5040**	**0.2127**	**−2.369**	**0.0178**
Pollination mode				
*Abiotic*				
Biotic	**−0.5216**	**0.2154**	**−2.421**	**0.0155**
Mixed (biotic and abiotic)	−0.1009	0.3176	−0.318	0.7508
(C) Species distribution range				
(Intercept)	0.1889	0.1422	1.328	0.1842
Distribution				
*Widespread*				
Restricted	−0.4200	0.2201	−1.909	0.0563

**TABLE 4:** Analysis of variance (ANOVA) testing the predictor variable effect on *Conservation status*, *Endemism*, and *Species Distribution Range* models. Chisq denotes the chi‐square statistic, df the degrees of freedom, and Pr(>Chisq) the associated *p‐*value. Pollination mode was significant (*p < 0.05) in both *Conservation status* and *Endemism* models, with *conservation status* and *endemism* also significant in their respective models.

Model	Predictor	Chisq	df	Pr(>Chisq)
Conservation status	Conservation status	4.360	1	0.03679*
	Pollination mode	6.882	2	0.03203*
Endemism	Endemism	5.6130	1	0.01783*
	Pollination mode	6.7063	2	0.03497*
Species distribution range	Species distribution range	3.6427	1	0.05632

## 4 Discussion

Assessing genetic diversity is mandatory to help predict population resilience and to guide conservation plans and management efforts, mainly for species on the brink of extinction such as rare, endemic, and threatened plant species presenting rare sexual systems. Whilst most conservation plans would benefit from assessments of intra‐ and interpopulation genetic diversity, this kind of information is still scarce and has been unevenly investigated in flowering plants worldwide (e.g., Essi et al. 2020; Exposito‐Alonso et al. 2022; Hamrick et al. 1991; Hogg et al. 2022; Kougioumoutzis et al. 2021; Lira 2024; López‐Pujol et al. 2009). Filling this biodiversity shortfall, attributed largely to the bias in funding support throughout the tree of life (Guénard et al. 2025), is nowadays paramount. Furthermore, broadening knowledge on evolutionary processes based on published available data is needed to delineate practical conservation actions. In this context, quantitative syntheses such as systematic reviews and meta‐analyses serve as a powerful tool for conservation science, as they can help to identify patterns and further knowledge gaps that require special attention (e.g., Clark and Pinsky 2024; De Kort et al. 2021; Figuerola‐Ferrando et al. 2023; Gamba and Muchhala 2020; González et al. 2020; He et al. 2024; Ohsawa and Ide 2008). Importantly, the outcomes of such studies may have direct conservation applications, guiding the prioritization of species for protection and supporting policy decisions in the absence of species‐specific genetic assessments.

Against this background, synthesizing genetic data specifically for dioecious flowering plants becomes particularly relevant. Dioecy is relatively rare among angiosperms, yet dioecious species are disproportionately represented among threatened taxa (K. S. Bawa 1980; Renner and Ricklefs 1995; Vamosi and Vamosi 2005). Although obligate outcrossing in dioecious systems is often expected to promote higher levels of genetic diversity, which may have a relevant impact on the extinction risk of populations, both theoretical and empirical studies suggest that several demographic and ecological constraints may limit these expected benefits.

To help bridge this knowledge gap, we conducted a quantitative synthesis of published genetic data for dioecious flowering plants to examine how ecological and life‐history traits shape patterns of intrapopulation genetic diversity in this group. Our results reveal that pollination mode plays a significant role in the patterns of intrapopulation genetic diversity among dioecious flowering plants, with biotically pollinated species exhibiting lowest genetic diversity. Endemism also emerged as an important predictor of intrapopulation genetic diversity, with populations of endemic species showing reduced levels of *H*_E_. Furthermore, conservation status was linked to the patterns of genetic diversity, aligning with the expected lower variability in threatened plant species (e.g., Spielman et al. 2004). Together, these results constitute a baseline to inform, guide, and establish conservation and management programs in attempting to safeguard dioecious flowering plants.

### 4.1 Depicting the Amount of *H*_E_ in Dioecious Plant Species

Theoretical advantages of dioecy in the origin and maintenance of genetic variation throughout allogamy seems not to warrant pronounced levels of genetic diversity between dioecious flowering plants. As a matter of fact, our analyses indicated that endemic and threatened dioecious flowering species had lowest levels of intrapopulation genetic diversity, reinforcing the well‐established link between genetic diversity loss and extinction risk (e.g., Boyd et al. 2022; Gitzendanner and Soltis 2000; Ishii et al. 2022; López‐Pujol et al. 2013; Samokhvalova 2022; Spielman et al. 2004; Tang et al. 2024; Vamosi and Vamosi 2005; but see Teixeira and Nazareno 2021; Turchetto et al. 2016). In spite of the pattern already documented in flowering plants (e.g., Hamrick and Godt 1990; Hamrick et al. 1992), narrow geographic distribution was not significantly linked to lower levels of intrapopulation genetic diversity in our dataset.

As endemism is commonly used to determine rarity (Rabinowitz 1981), and considering the negative effects of it on intrapopulation genetic diversity, we stress that genetic diversity parameters such as expected heterozygosity should be included as one of the elemental indicators to characterize rare species. Not only focused on rare plant species with uncommon sexual systems, it would be pivotal in conservation agendas as a consequence. In addition to conservation‐related predictors, our study demonstrates the relevance of pollination mode as a predictor of genetic diversity in dioecious angiosperms. How pollination mode acts on dioecious plant species have attracted research interest so far. While some studies suggested an association between dioecy and pollination by small, unspecialized insects (e.g., K. S. Bawa 1994; Bawa and Opler 1975; Ibarra‐Manríquez and Oyama 1992), a stronger association between dioecy and abiotic pollination modes (e.g., anemophily, hydrophily) has often been noticed, particularly by anemophily (e.g., Darwin 1876; Kerner Von Mariaun 1895; Renner and Feil 1993; Sporne 1949; Stebbins 1951; Steiner 1988) which estimate bears 30% (Renner 2014). Besides, many entomophilous plant species exhibiting mixed pollination mode have employed anemophily as a secondary pollination strategy (Abrahamczyk et al. 2023; Renner and Feil 1993). It is noteworthy that, in contrast to pollination patterns reported in systematic studies (e.g., Bullock 1994; Renner 2014; Vamosi et al. 2003), biotically pollinated species are overrepresented in our dataset, an Eltonian shortfall to be further attenuated. Taking into account that we clustered all forms of animal‐mediated pollination into a single category, this aggregation may partly contribute to the predominance of biotic pollination. Nevertheless, this pattern may also reflect broader biases in the availability of genetic studies (Ballesteros‐Mejia et al. 2016). For instance, several dioecious tropical trees are wind‐pollinated (Bullock 1994; Renner 2014; Vamosi et al. 2003), yet tropical plant species remain underrepresented in many ecological and genetic syntheses due to persistent geographic and taxonomic research biases (Hortal et al. 2015; Linck and Cadena 2025).

Our findings indicate that dioecious flowering species relying on biotic pollination harbor lower levels of intrapopulation genetic diversity compared to those abiotically pollinated. Whilst biotic pollination (i.e., animal‐mediated) may enhance genetic connectivity through targeted and occasionally long‐distance pollen transfer (Aranda‐Rickert et al. 2021; Levin and Kerster 1974), it is inherently more vulnerable to failure than abiotic pollination modes (e.g., anemophily, hydrophily; Friedman and Barrett 2009; Kevan and Viana 2003; Maciel et al. 2020). This pattern likely reflects the intrinsic biological susceptibility of dioecious flowering plants to environmental and climate change driven by human economic activities, impacting population demography (i.e., biased sexual ratio), modifying phenological patterns, reducing effective population sizes, intensifying genetic drift and biparental inbreeding, and disrupting plant‐pollinator interactions as a consequence (e.g., Ashworth et al. 2004; de Jesus Aguilar‐Aguilar et al. 2023; Didham et al. 1996; Fisogni et al. 2025; Goverde et al. 2002; Hegland et al. 2009; Vranckx et al. 2012). With forest conversion rates scaling up (Lapola et al. 2023; Schauman et al. 2024), especially in the tropics (Ma et al. 2023), the conservation of plant species with rare sexual system such as dioecy is urgently needed (Heilbuth 2000; Renner and Ricklefs 1995; Vamosi et al. 2003).

### 4.2 Limitations and Caveats

While our study identified key life‐history traits as predictors of genetic diversity in dioecious flowering plants, a substantial portion of the variance remained unexplained. Future research integrating ecological and demographic factors such as landscape metrics, population effective size, spatial genetic structure, plant density, and population sex ratio, which may contribute to modulate the levels of genetic diversity, should be done particularly considering the unique reproductive constraints of dioecy.

Although sex ratio and effective population size may act as predictors and help to puzzle the genetic diversity pattern on dioecious plants, such data is still scarce and were only reported in few of the studies analyzed (i.e., Bard et al. 2021; Burge 2020; Cascante‐Marín et al. 2020; Chavez‐Pesqueira et al. 2014; Ci et al. 2008; de Jesus Aguilar‐Aguilar et al. 2023; Gustafson et al. 2013; Lauterbach et al. 2012; Magalhaes et al. 2011; Mitchell et al. 1999; Nakamura et al. 2021; Rosche et al. 2018; Rottenberg and Parker 2003; Rottenberg et al. 1999; Segarra‐Moragues et al. 2005; Walcott et al. 2025; Zhao et al. 2010). For instance, from 62 published articles addressed in our review, nine presented estimates of population sizes (i.e., Bard et al. 2021; Burge 2020; Chavez‐Pesqueira et al. 2014; Ci et al. 2008; Lauterbach et al. 2012; Magalhaes et al. 2011; Mitchell et al. 1999; Walcott et al. 2025; Zhao et al. 2010) and eleven discussed the sex ratios within each sampled location (i.e., Burge 2020; Cascante‐Marín et al. 2020; de Jesus Aguilar‐Aguilar et al. 2023; Gustafson et al. 2013; Lauterbach et al. 2012; Nakamura et al. 2021; Rottenberg and Parker 2003; Rottenberg et al. 1999; Rosche et al. 2018; Segarra‐Moragues et al. 2005; Walcott et al. 2025). New empirical studies should consider and discuss these features whenever possible, given the importance of demographic and phenological factors for the population dynamics of dioecious flowering plants. Nevertheless, collecting samples during specific flowering and fruiting periods is often unfeasible, as these events do not occur simultaneously in all individuals. Another challenge is to identify the sex of non‐reproductive individuals in the field, requiring the development and use of molecular methods, which are unavailable for the majority of the plant species, particularly for the rare and threatened ones.

Another relevant point is that our models do not account for the evolutionary history of the species included in the dataset. In fact, factors such as divergence time, past demographic events (e.g., population bottlenecks or expansions), historical climatic fluctuations, or lineage‐specific mutation rates, may all contribute to the intrapopulation genetic structure of such species (Hamrick et al. 1981; Pfenninger et al. 2011). For example, species with older evolutionary origins may have had more time to accumulate mutations; however, such variation could also have been reduced or lost due to historical demographic contractions. These evolutionary and historical factors, which were not explicitly modeled here, may therefore partially explain the residual variation observed in our analyses. Moreover, although some of these attributes may have been represented by the random effect “species”, which explained a large proportion of the variance in the models, further investigation into how evolutionary history shapes genetic diversity in dioecious flowering plants would be highly valuable, particularly in a conservation context.

## Author Contributions

**Thais Martins Teixeira:** conceptualization (equal), data curation (lead), formal analysis (lead), investigation (lead), methodology (equal), writing – original draft (lead), writing – review and editing (equal). **Alison Gonçalves Nazareno:** conceptualization (equal), formal analysis (supporting), investigation (supporting), methodology (equal), supervision (lead), writing – original draft (supporting), writing – review and editing (equal).

## Conflicts of Interest

The authors declare no conflicts of interest.

## Supporting information


**Appendix S1:** ece373459‐sup‐0001‐TableS1‐S2‐FigureS1‐S10.docx.

**Data S1:** ece373459‐sup‐0002‐supinfo.xlsx.

## Data Availability

The data that supports the findings of this study are available in the Supporting Information of this article.
