# Supplementary material for: Separated Sexes, Shared Consequences: Disentangling Predictors of Genetic Diversity in Dioecious Angiosperms
Source: Ecol Evol. 2026 May 10;16(5):e73459. doi: 10.1002/ece3.73459 (PMC13158526; doi:10.1002/ece3.73459)
Supplement: Supplementary file 1 — Appendix S1: ece373459‐sup‐0001‐TableS1‐S2‐FigureS1‐S10.docx. [file ECE3-16-e73459-s001.docx]

# **SEPARATED SEXES, SHARED CONSEQUENCES: DISENTANGLING PREDICTORS OF GENETIC DIVERSITY IN DIOECIOUS ANGIOSPERMS**

THAIS MARTINS TEIXEIRA AND ALISON GONÇALVES NAZARENO

**SUPPORTING INFORMATION**

**TABLE S1** Taxonomic changes within the species sampled for a review of the patterns of genetic diversity in dioecious flowering plants based on the most recent updates of GBIF Taxonomic [Backbone](https://www.sciencedirect.com/topics/agricultural-and-biological-sciences/spine) and Plants of World Online (POWO) databases. The taxonomic changes are highlighted in bold.

| **Article classification** | | | **Taxonomic updates (GBIF / POWO)** | | |
| --- | --- | --- | --- | --- | --- |
| **Species** | **Genus** | **Family** | **Species / syn.** | **Genus** | **Family** |
| *Myrica faya* | Myrica | Myricaceae | ***Morella*** *faya* | **Morella** | Myricaceae |
| *Myrica rivas-martinezii* | Myrica | Myricaceae | ***Morella*** *rivas-martinezii* | **Morella** | Myricaceae |
| *Rumex rothschildianus* | Rumex | Polygonaceae | *Rumex* ***aeroplaniformis*** | Rumex | Polygonaceae |
| *Asparagus acutifolius* | Asparagus | Liliaceae | *Asparagus acutifolius* | Asparagus | **Asparagaceae** |
| *Empetrum nigrum var. japonicum* | Empetrum | Empetraceae | *Empetrum nigrum var. japonicum* | Empetrum | **Ericaceae** |
| *Eurya spp.* | Eurya | Theaceae | *Eurya spp.* | Eurya | **Pentaphylacaceae** |
| *Uapaca kirkiana* | Uapaca | Euphorbiaceae | *Uapaca kirkiana* | Uapaca | **Phyllanthaceae** |
| *Kmeria septentrionalis* | Kmeria | Magnoliacea | ***Magnolia*** *kwangsiensis* | **Magnolia** | Magnoliacea |
| *Borderea chouardii* | *Borderea* | Dioscoreaceae | [***Dioscorea*** *chouardii*](https://powo.science.kew.org/taxon/urn:lsid:ipni.org:names:317804-1) | **Dioscorea** | Dioscoreaceae |

**TABLE S2** Description of models with AIC > 4 resulted from the dredge function applied to the three comprehensive Generalized Linear Mixed Models (Conservation status, Endemism and Species distribution range) for intrapopulation genetic diversity of dioecious flowering plants. Models with the lower AICc are showed in bold.

| **Model predictors** | **df** | **logLik** | **AICc** | **delta** | **weight** |
| --- | --- | --- | --- | --- | --- |
| ***Conservation status*** | | | | | |
| **Pollination mode + Conservation status** | **8** | **164.84** | **-313.42** | **0.00** | **0.12** |
| Seed dispersal syndrome + Pollination mode + Conservation status | 10 | 166.65 | -312.92 | 0.50 | 0.09 |
| Growth form + Conservation status | 7 | 163.55 | -312.90 | 0.52 | 0.09 |
| Conservation status | 5 | 161.48 | -312.86 | 0.56 | 0.09 |
| Growth form + Pollination mode + Conservation status | 10 | 166.48 | -312.58 | 0.84 | 0.08 |
| Seed dispersal syndrome + Conservation status | 8 | 164.12 | -312.00 | 1.43 | 0.06 |
| Pollination mode + Reproductive mode + Conservation status | 9 | 164.87 | -311.42 | 2.00 | 0.04 |
| Growth form + Reproductive mode + Conservation status | 8 | 163.81 | -311.36 | 2.06 | 0.04 |
| Pollination mode | 7 | 162.67 | -311.15 | 2.27 | 0.04 |
| Null model | 4 | 159.60 | -311.13 | 2.29 | 0.04 |
| Growth form + Pollination mode + Reproductive mode + Conservation status | 11 | 166.72 | -310.97 | 2.45 | 0.03 |
| Seed dispersal syndrome + Pollination mode + Reproductive mode + Conservation status | 11 | 166.72 | -310.97 | 2.45 | 0.03 |
| Reproductive mode + Conservation status | 6 | 161.52 | -310.90 | 2.52 | 0.03 |
| Seed dispersal syndrome + Growth form + Pollination mode + Conservation status | 12 | 167.58 | -310.61 | 2.81 | 0.03 |
| Seed dispersal syndrome + Reproductive mode + Conservation status | 9 | 164.19 | -310.07 | 3.36 | 0.02 |
| Seed dispersal syndrome + Growth form + Conservation status | 10 | 165.12 | -309.85 | 3.57 | 0.02 |
| Growth form | 6 | 160.95 | -309.75 | 3.67 | 0.02 |
|  |  |  |  |  |  |
| ***Endemism*** | | | | | |
| **Endemism + Pollination mode** | **8** | **165.39** | **-314.54** | **0.00** | **0.15** |
| Seed dispersal syndrome + Endemism + Pollination mode | 10 | 167.25 | -314.11 | 0.43 | 0.12 |
| Endemism | 5 | 161.81 | -313.51 | 1.02 | 0.09 |
| Seed dispersal syndrome + Endemism | 8 | 164.82 | -313.39 | 1.15 | 0.09 |
| Endemism + Growth form + Pollination mode | 10 | 166.63 | -312.88 | 1.66 | 0.07 |
| Endemism + Growth form | 7 | 163.46 | -312.72 | 1.82 | 0.06 |
| Endemism + Pollination mode + Reproductive mode | 9 | 165.45 | -312.58 | 1.96 | 0.06 |
| Seed dispersal syndrome + Endemism + Pollination mode + Reproductive mode | 11 | 167.29 | -312.12 | 2.42 | 0.05 |
| Endemism + Reproductive mode | 6 | 161.82 | -311.50 | 3.04 | 0.03 |
| Seed dispersal syndrome + Endemism + Reproductive mode | 9 | 164.86 | -311.41 | 3.13 | 0.03 |
| Pollination mode | 7 | 162.67 | -311.15 | 3.39 | 0.03 |
| Null model | 4 | 159.60 | -311.13 | 3.40 | 0.03 |
| Seed dispersal syndrome + Endemism + Growth form + Pollination mode | 12 | 167.76 | -310.97 | 3.56 | 0.03 |
| Endemism + Growth form + Pollination mode + Reproductive mode | 11 | 166.64 | -310.80 | 3.73 | 0.02 |
| Endemism + Growth form + Reproductive mode | 8 | 163.48 | -310.70 | 3.84 | 0.02 |
|  |  |  |  |  |  |
| ***Species distribution range*** | | | | | |
| **Seed dispersal syndrome + Species distribution range** | **8** | **164.61** | **-312.96** | **0.00** | **0.10** |
| Species distribution range + Growth form | 7 | 163.50 | -312.80 | 0.16 | 0.10 |
| Species distribution range | 5 | 161.39 | -312.67 | 0.30 | 0.09 |
| Seed dispersal syndrome + Species distribution range + Pollination mode | 10 | 166.40 | -312.42 | 0.55 | 0.08 |
| Species distribution range + Pollination mode | 8 | 164.29 | -312.33 | 0.63 | 0.08 |
| Species distribution range + Growth form + Pollination mode | 10 | 165.95 | -311.52 | 1.45 | 0.05 |
| Species distribution range + Growth form + Reproductive mode | 8 | 163.86 | -311.47 | 1.49 | 0.05 |
| Pollination mode | 7 | 162.67 | -311.15 | 1.82 | 0.04 |
| Null model | 4 | 159.60 | -311.13 | 1.83 | 0.04 |
| Seed dispersal syndrome + Species distribution range + Reproductive mode | 9 | 164.70 | -311.07 | 1.89 | 0.04 |
| Seed dispersal syndrome + Species distribution range + Growth form | 10 | 165.60 | -310.82 | 2.15 | 0.04 |
| Species distribution range + Reproductive mode | 6 | 161.46 | -310.77 | 2.20 | 0.03 |
| Seed dispersal syndrome + Species distribution range + Pollination mode + Reproductive mode | 11 | 166.48 | -310.49 | 2.48 | 0.03 |
| Species distribution range + Pollination mode + Reproductive mode | 9 | 164.33 | -310.35 | 2.62 | 0.03 |
| Seed dispersal syndrome + Species distribution range + Growth form + Pollination mode | 12 | 167.28 | -310.01 | 2.96 | 0.02 |
| Species distribution range + Growth form + Pollination mode + Reproductive mode | 11 | 166.22 | -309.96 | 3.00 | 0.02 |
| Growth form | 6 | 160.95 | -309.75 | 3.21 | 0.02 |
| Seed dispersal syndrome + Species distribution range + Growth form + Reproductive mode | 11 | 165.89 | -309.31 | 3.65 | 0.02 |
| Reproductive mode | 5 | 159.69 | -309.28 | 3.69 | 0.02 |
| Pollination mode + Reproductive mode | 8 | 162.74 | -309.22 | 3.74 | 0.02 |
| Growth form + Pollination mode | 9 | 163.74 | -309.16 | 3.80 | 0.02 |
| Seed dispersal syndrome + Pollination mode | 9 | 163.71 | -309.11 | 3.85 | 0.02 |


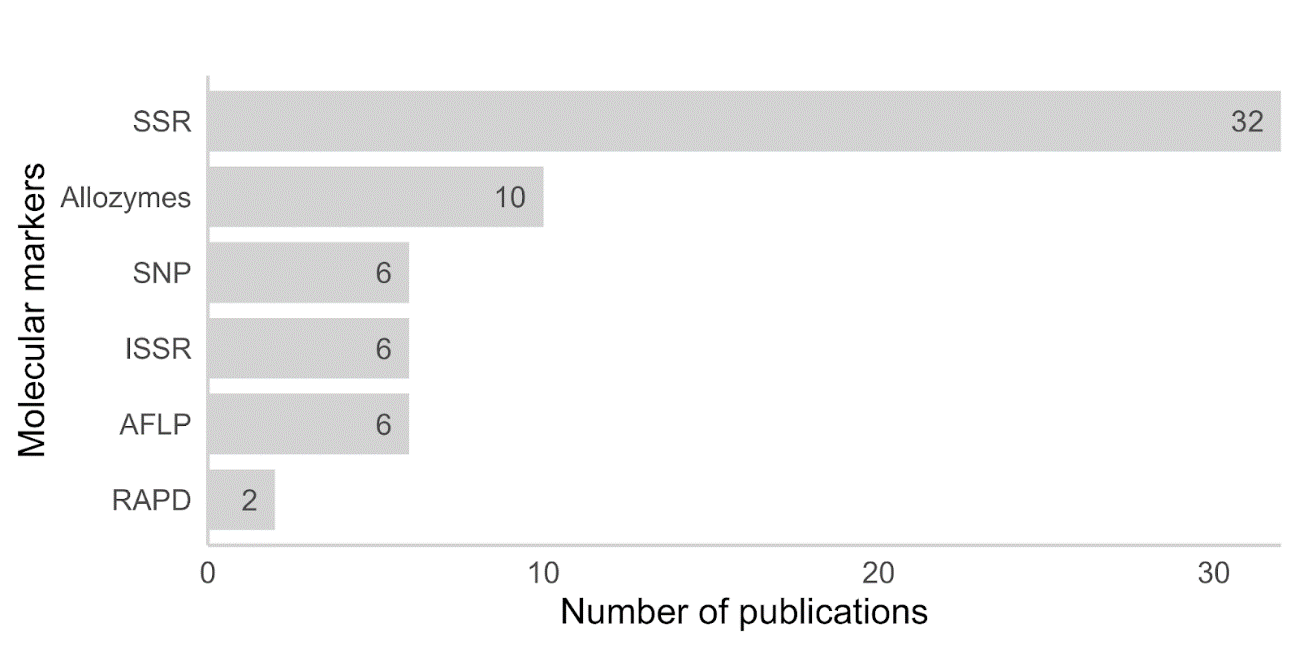


**FIGURE S1** Relative frequency of each molecular marker (Allozymes; AFLP, Amplified Fragment Length Polymorphism; ISSR, Inter Simple Sequence Repeats; SSR, Simple Sequence Repeat; SNP, Single Nucleotide Polymorphism; RAPD, Random Amplified Polymorphic DNA) used to estimate the genetic diversity parameter (i.e., expected heterozygosity, *H*_E_) in 66 dioecious flowering plants based on 62 published studies.


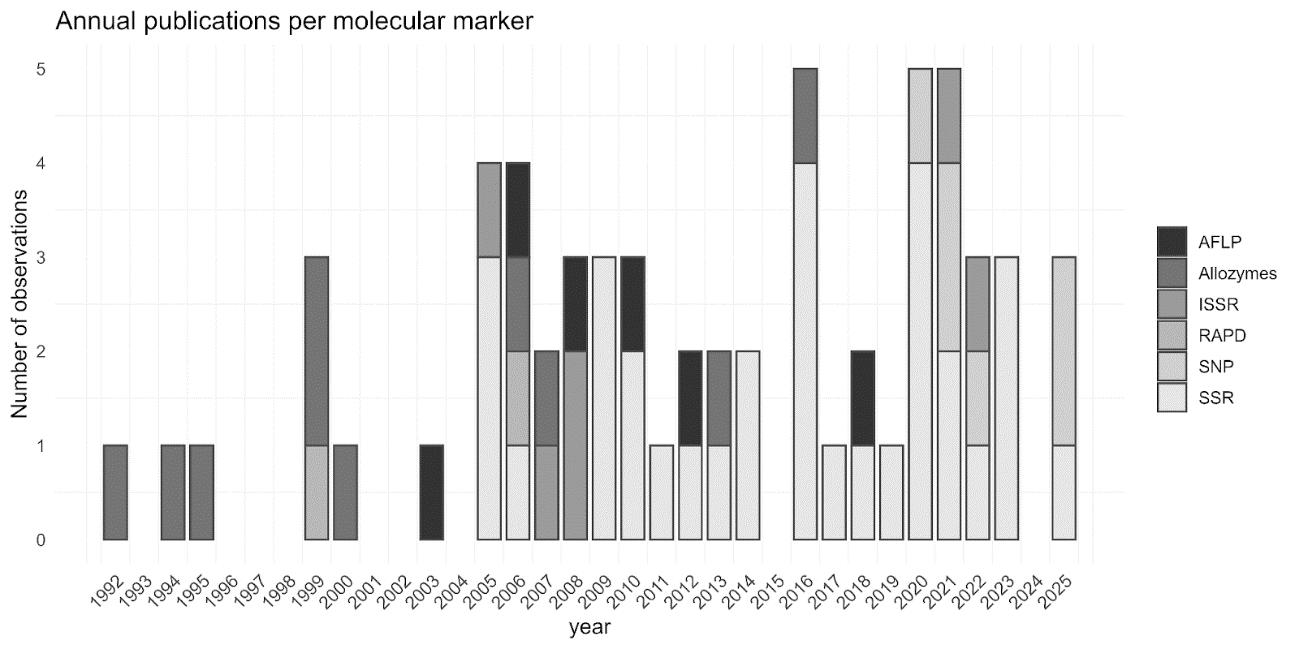


**FIGURE S2** Summary of the proportion of each molecular marker in the scientific publications per year included in the dataset of population genetics of dioecious flowering plants. The colors represent different markers, while the bars’ height represent the number of publications in each year, showing an irregular pattern of research output over time, with peaks observed between 2010 and 2022. Notably, isozymes dominated the field until 2000, while SSR markers have seen a progressive increase in use from 2005 to the present. Since 2020 the use of SNP markers is becoming frequent, which is associated with the emergence of high throughout sequencing technologies.

| 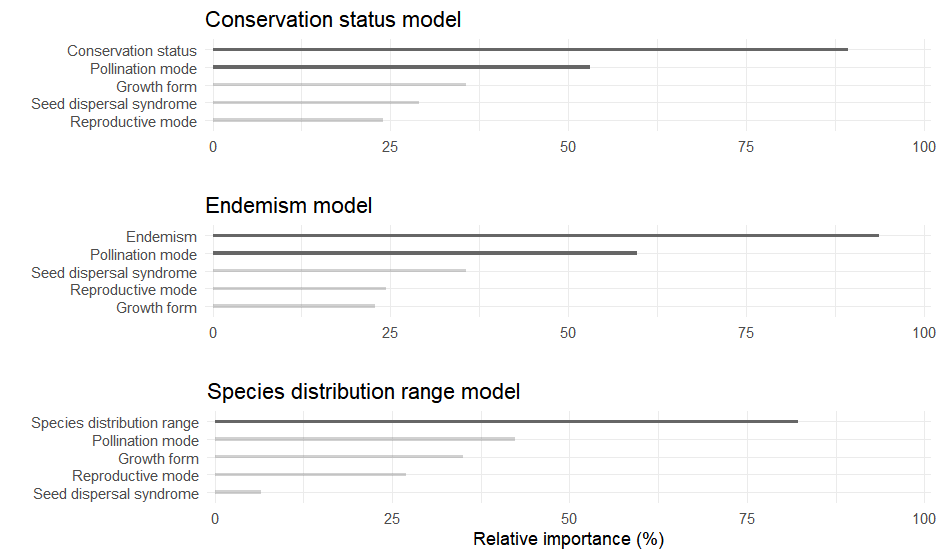 |
| --- |

**FIGURE S3** Relative importance (RI) of predictor variables in three comprehensive models evaluating separately the effects of conservation status, species distribution range, and endemism, with growth form, pollination mode, seed dispersal syndrome, and reproductive mode as additional predictors of intrapopulational genetic diversity of dioecious flowering plants. Transparent lines indicate predictors RI < 50%, and therefore were not retained in the respective final Generalized Linear Mixed Model.


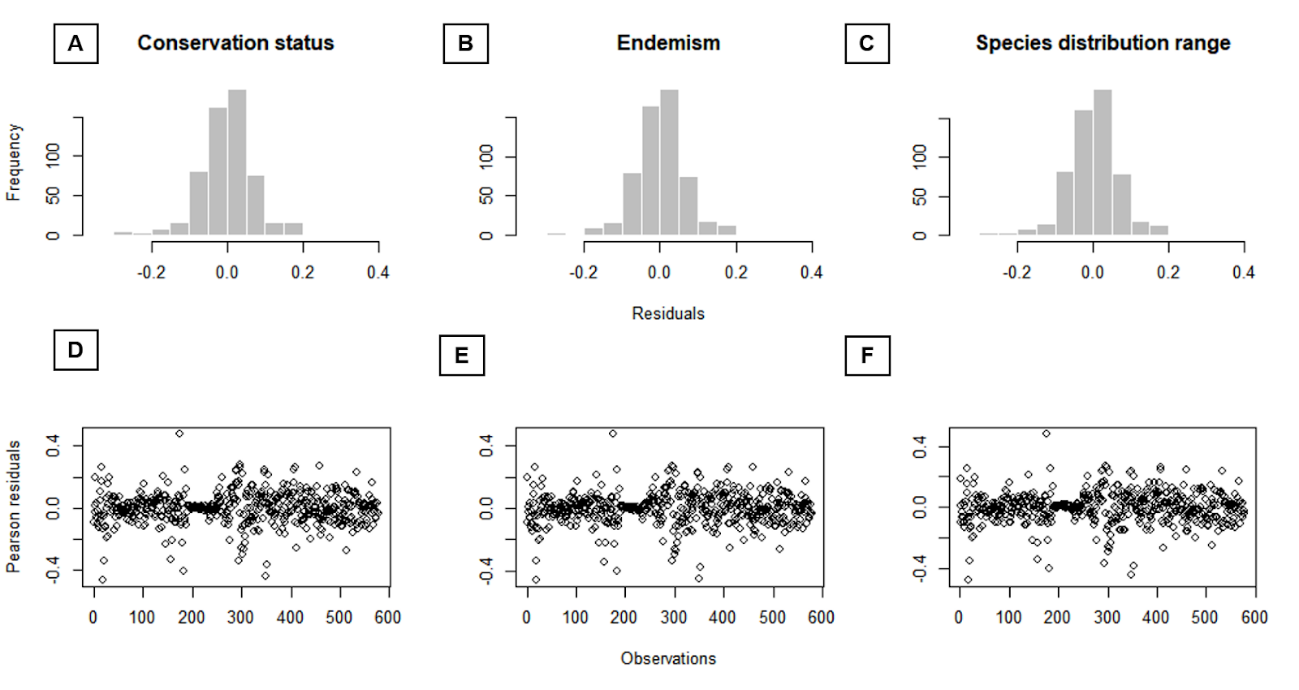


**FIGURE** **S4** Histograms (A-C) and Pearson' residuals (D-F) are presented for each Generalized Linear Mixed Models (i.e., Conservation Status, Endemism, Species Distribution Range) and were computed based on intrapopulation genetic diversity (i.e., expected heterozygosity) of 66 dioecious flowering plants. Pearson’ residuals were approximately centered around zero with no visible pattern across fitted values, indicating no apparent heteroscedasticity.

**References of the papers included in the dataset of intrapopulation genetic diversity of dioecious angiosperms**

Abarca, C. A., Martinez-Bauer, A., Molina-Freaner, F., & Dominguez, C. A. (2008). The genetic consequences of evolving two sexes:: the genetic structure of distylous and dioecious species of *Erythroxylum*. *Evolutionary Ecology Research*, *10*(2), 281–293. https://www.evolutionary-ecology.com/issues/v10n02/kkar2256.pdf

Aguiar, B. I., Sebbenn, A. M., Tarazi, R., Vogado, N. O., Morellato, L. P. C., Tambarussi, E. V., Moreno, M. A., Pereira, L. C. S. M., Montibeller, C., Ferraz, E. M., Gandara, F. B., & Kageyama, P. Y. (2020). Phenology, Seed Germination, and Genetics Explains the Reproductive Strategies of *Diospyros lasiocalyx* (Mart.) B. Wall. *Tropical Plant Biology*, *13*(1), 23–35. https://doi.org/10.1007/s12042-019-09243-1

Bard, N. W., Miller, C. S., & Bruederle, L. P. (2021). High genomic diversity maintained by populations of *Carex scirpoidea* subsp. *convoluta*, a paraphyletic Great Lakes ecotype. *Conservation Genetics*, *22*(2), 169–185. https://doi.org/10.1007/s10592-020-01326-x

Bartish, Jeppsson, & Nybom. (1999). Population genetic structure in the dioecious pioneer plant species *Hippophae rhamnoides* investigated by random amplified polymorphic DNA (RAPD) markers. *Molecular Ecology*, *8*(5), 791–802. https://doi.org/10.1046/j.1365-294X.1999.00631.x

Bizoux, J. ‐P., Daïnou, K., Bourland, N., Hardy, O. J., Heuertz, M., Mahy, G., & Doucet, J. ‐L. (2009). Spatial genetic structure in *Milicia excelsa* (Moraceae) indicates extensive gene dispersal in a low‐density wind‐pollinated tropical tree. *Molecular Ecology*, *18*(21), 4398–4408. https://doi.org/10.1111/j.1365-294X.2009.04365.x

Brown, J. E., Bauman, J. M., Lawrie, J. F., Rocha, O. J., & Moore, R. C. (2012). The Structure of Morphological and Genetic Diversity in Natural Populations of *Carica papaya* (Caricaceae) in Costa Rica. *Biotropica*, *44*(2), 179–188. https://doi.org/10.1111/j.1744-7429.2011.00779.x

Burge, D. (2020). Conservation genomics and pollination biology of an endangered, edaphic-endemic, octoploid herb: El Dorado bedstraw (*Galium californicum* subsp. *sierrae* ; Rubiaceae). *PeerJ*, *8*, e10042. https://doi.org/10.7717/peerj.10042

Cascante-Marín, A., Trejos, C., Madrigal, R., & Fuchs, E. J. (2020). Genetic diversity and reproductive biology of the dioecious and epiphytic bromeliad *Aechmea mariae-reginae* (Bromeliaceae) in Costa Rica: implications for its conservation. *Botanical Journal of the Linnean Society*, *192*(4), 773–786. https://doi.org/10.1093/botlinnean/boz083

Chávez‐Pesqueira, M., Suárez‐Montes, P., Castillo, G., & Núñez‐Farfán, J. (2014). Habitat fragmentation threatens wild populations of *Carica papaya* (Caricaceae) in a lowland rainforest. *American Journal of Botany*, *101*(7), 1092–1101. https://doi.org/10.3732/ajb.1400051

Chen, G., Wang, Y., Zhao, C., Korpelainen, H., & Li, C. (2008). Genetic diversity of *Hippophae rhamnoides* populations at varying altitudes in the Wolong Natural Reserve of China as revealed by ISSR markers. *Silvae Genetica*, *57*(1), 29 – 36. https://doi.org/10.1515/sg-2008-0005

Chung, M. G., & Kang, S. S. (1994). Genetic-variation and population-structure in korean populations Of *Eurya japonica* (Theaceae). *AMERICAN JOURNAL OF BOTANY*, *81*(8), 1077–1082. https://doi.org/10.2307/2445303

Chung, M. G., & Kang, S. S. (1995). Allozyme diversity and genetic structure in Korean populations of *Eurya emaginata* (Theaceae). *Japanese Journal of Genetics*, *70*(3), 387 – 398. https://doi.org/10.1266/jjg.70.387

Chung, M. Y., López-Pujol, J., Moon, M.-O., Chung, J. M., Kim, C. S., Sun, B.-Y., Kim, K.-J., & Chung, M. G. (2013). Comparison of genetic diversity in the two arctic-alpine plants *Diapensia lapponica* var. *obovata* (Diapensiaceae) and *Empetrum nigrum* var. *japonicum* (Empetraceae) between Sakhalin in Russian Far East and Jeju Island in Korea, the southernmost edge of their distribution range. *Population Ecology*, *55*(1), 159 – 172. https://doi.org/10.1007/s10144-012-0348-z

Ci, X., Chen, J., Li, Q., & Li, J. (2008). AFLP and ISSR analysis reveals high genetic variation and inter-population differentiation in fragmented populations of the endangered *Litsea szemaois* (Lauraceae) from Southwest China. *Plant Systematics and Evolution*, *273*(3–4), 237–246. https://doi.org/10.1007/s00606-008-0012-4

Conroy, G. C., Shimizu-Kimura, Y., Lamont, R. W., & Ogbourne, S. M. (2019). A multidisciplinary approach to inform assisted migration of the restricted rainforest tree, *Fontainea rostrata*. *PLOS ONE*, *14*(1), e0210560. https://doi.org/10.1371/journal.pone.0210560

Cristóbal-Pérez, E. J., Fuchs, E. J., Martén-Rodríguez, S., & Quesada, M. (2021). Habitat fragmentation negatively affects effective gene flow via pollen, and male and female fitness in the dioecious tree, *Spondias purpurea* (Anacardiaceae). *Biological Conservation*, *256*, 109007. https://doi.org/10.1016/j.biocon.2021.109007

Cristóbal-Pérez, E. J., Fuchs, E. J., Olivares-Pinto, U., & Quesada, M. (2020). Janzen-Connell effects shape gene flow patterns and realized fitness in the tropical dioecious tree *Spondias purpurea* (ANACARDIACEAE). *Scientific Reports*, *10*(1), 4584. https://doi.org/10.1038/s41598-020-61394-4

Daïnou, K., Bizoux, J.-P., Doucet, J.-L., Mahy, G., Hardy, O. J., & Heuertz, M. (2010). Forest refugia revisited: nSSRs and cpDNA sequences support historical isolation in a wide-spread African tree with high colonization capacity, *Milicia excelsa* (Moraceae). *Molecular Ecology*, *19*(20), 4462–4477. https://doi.org/10.1111/j.1365-294X.2010.04831.x

De Cauwer, I., Delle‐Vedove, R., Buatois, B., Godé, C., & Dufay, M. (2025). Striking variation of pollinator attracting scent within a highly specialized pollination system. *Journal of Ecology*, *113*(4), 856–868. https://doi.org/10.1111/1365-2745.14493

de Jesus Aguilar‐Aguilar, M., Cristobal‐Pérez, E. J., Lobo, J., Fuchs, E. J., Oyama, K., Martén‐Rodríguez, S., Herrerías‐Diego, Y., & Quesada, M. (2023). Gone with the wind: Negative genetic and progeny fitness consequences of habitat fragmentation in the wind pollinated dioecious tree *Brosimum alicastrum*. *American Journal of Botany*, *110*(4). <https://doi.org/10.1002/ajb2.16157>

de Oliveira Melo, A. T. de O., & Franceschinelli, E. V. (2016). Gene flow and fine-scale spatial genetic structure in *Cabralea canjerana* (Meliaceae), a common tree species from the Brazilian Atlantic forest. *Journal of Tropical Ecology*, *32*(2), 135–145. https://doi.org/10.1017/S0266467416000067

Ferrer, M. M., Tapia-Gómez, C. A., Estrada-Medina, H., Ruenes-Morales, M. del R., Montañez-Escalante, P. I., & Jiménez-Osornio, J. J. (2021). Growing Out of the Tropical Forests: Gene Flow of Native Mesoamerican Trees Among Forest and Mayan Homegardens. *Frontiers in Ecology and Evolution*, *9*. https://doi.org/10.3389/fevo.2021.628765

Feyissa, T., Nybom, H., Bartish, I. V., & Welander, M. (2007). Analysis of genetic diversity in the endangered tropical tree species *Hagenia abyssinica* using ISSR markers. *Genetic Resources and Crop Evolution*, *54*(5), 947–958. https://doi.org/10.1007/s10722-006-9155-8

Fuchs, E. J., Cascante-Marín, A., Madrigal-Brenes, R., & Quesada, M. (2023). Genetic diversity and phylogeographic patterns of the dioecious palm *Chamaedorea tepejilote* (Arecaceae) in Costa Rica: the role of mountain ranges and possible refugia. *AoB PLANTS*, *15*(1). https://doi.org/10.1093/aobpla/plac060

González-Pérez, M. A., Sosa, P. A., Rivero, E., González-González, E. A., & Naranjo, A. (2009). Molecular markers reveal no genetic differentiation between *Myrica rivas-martinezii* and *M. faya* (Myricaceae). *Annals of Botany*, *103*(1), 79–86. https://doi.org/10.1093/aob/mcn222

Gustafson, D. J., Giunta, A. P., & Echt, C. S. (2013). Extensive clonal growth and biased sex ratios of an endangered dioecious shrub, *Lindera melissifolia* (Walt) Blume (Lauraceae) ^1^. *The Journal of the Torrey Botanical Society*, *140*(2), 133–144. https://doi.org/10.3159/TORREY-D-12-00072.1

Huang, J.-F., Li, S.-Q., Xu, R., & Peng, Y.-Q. (2023). East‒West genetic differentiation across the Indo-Burma hotspot: evidence from two closely related dioecious figs. *BMC Plant Biology*, *23*(1), 321. https://doi.org/10.1186/s12870-023-04324-6

Lamont, R. W., Conroy, G. C., Reddell, P., & Ogbourne, S. M. (2016). Population genetic analysis of a medicinally significant Australian rainforest tree, *Fontainea picrosperma* C.T. White (Euphorbiaceae): biogeographic patterns and implications for species domestication and plantation establishment. *BMC Plant Biology*, *16*(1), 57. https://doi.org/10.1186/s12870-016-0743-2

Lauterbach, D., Ristow, M., & Gemeinholzer, B. (2012). Population genetics and fitness in fragmented populations of the dioecious and endangered *Silene otites* (Caryophyllaceae). *Plant Systematics and Evolution*, *298*(1), 155–164. https://doi.org/10.1007/s00606-011-0533-0

Lin, J., Gibbs, J. P., & Smart, L. B. (2009). Population genetic structure of native versus naturalized sympatric shrub willows ( *Salix* ; Salicaceae). *American Journal of Botany*, *96*(4), 771–785. https://doi.org/10.3732/ajb.0800321

Luna, R., Epperson, B. K., & Oyama, K. (2007). High levels of genetic variability and inbreeding in two Neotropical dioecious palms with contrasting life histories. *Heredity*, *99*(4), 466–476. https://doi.org/10.1038/sj.hdy.6801027

Magalhaes, I. S., Gleiser, G., Labouche, A.-M., & Bernasconi, G. (2011). Comparative population genetic structure in a plant-pollinator/seed predator system. *Molecular Ecology*, *20*(22), 4618–4630. https://doi.org/10.1111/j.1365-294X.2011.05296.x

Manoel, R. O., Rossini, B. C., Cornacini, M. R., Moraes, M. L. T., Cambuim, J., Alcântara, M. A. M., Silva, A. M., Sebbenn, A. M., & Marino, C. L. (2021). Landscape barriers to pollen and seed flow in the dioecious tropical tree *Astronium fraxinifolium* in Brazilian savannah. *PLOS ONE*, *16*(8), e0255275. https://doi.org/10.1371/journal.pone.0255275

Mitchell, A., Hogan, K., & Chapman, H. (1999). Genetic variation in *Aciphylla glaucescens* (Apiaceae). *New Zealand Journal of Ecology*, *23*(1), 61 – 67.

Mooney, E., Edwards, M., & Niesenbaum, R. (2010). Genetic differentiation between sun and shade habitats in populations of *Lindera benzoin* L. *Population Ecology*, *52*(3), 417 – 425. https://doi.org/10.1007/s10144-010-0197-6

Morgan, E. J., Kaiser-Bunbury, C. N., Edwards, P. J., Fleischer-Dogley, F., & Kettle, C. J. (2017). Keeping it in the family: strong fine-scale genetic structure and inbreeding in *Lodoicea maldivica*, the largest-seeded plant in the world. *Conservation Genetics*, *18*(6), 1317 – 1329. https://doi.org/10.1007/s10592-017-0982-2

Mwase, W. F., Bjørnstad, Å., Stedje, B., Bokosi, J. M., & Kwapata, M. B. (2006). Genetic diversity of *Uapaca kirkiana* Muel. Årg. populations as revealed by amplified fragment length polymorphisms (AFLPs). *African Journal of Biotechnology*, *5*(13), 1205 – 1213.

Nakamura, M., Nanami, S., Okuno, S., Hirota, S. K., Matsuo, A., Suyama, Y., Tokumoto, H., Yoshihara, S., & Itoh, A. (2021). Genetic diversity and structure of apomictic and sexually reproducing *Lindera* species (Lauraceae) in Japan. *Forests*, *12*(2), 1 – 13. https://doi.org/10.3390/f12020227

Obbard, D. J., Harris, S. A., & Pannell, J. R. (2006). Sexual systems and population genetic structure in an annual plant: Testing the metapopulation model. *American Naturalist*, *167*(3), 354 – 366. https://doi.org/10.1086/499546

Peñaloza-Ramírez, J. M., Aguilar-Amezquita, B., Núñez-Farfán, J., Pérez-Nasser, N., Albarrán-Lara, A. L., & Oyama, K. (2016). Consequences of habitat fragmentation on genetic structure of *Chamaedorea alternans* (Arecaceae) palm populations in the tropical rain forests of Los Tuxtlas, Veracruz, Mexico. *Revista Mexicana de Biodiversidad*, *87*(3), 990–1001. https://doi.org/10.1016/j.rmb.2016.07.004

Ramadoss, N., Steele, S., & Flores‐Renteria, L. (2025). Prickly Problems: *Cylindropuntia* ’s Low Genetic Diversity Despite Inbreeding Avoidance. *Ecology and Evolution*, *15*(4). https://doi.org/10.1002/ece3.71213

Rosche, C., Schrieber, K., Lachmuth, S., Durka, W., Hirsch, H., Wagner, V., Schleuning, M., & Hensen, I. (2018). Sex ratio rather than population size affects genetic diversity in *Antennaria dioica*. *Plant Biology*, *20*(4), 789–796. https://doi.org/10.1111/plb.12716

Rottenberg, A., Nevo, E., & Zohary, D. (2000). Genetic variability in sexually dimorphic and monomorphic populations of *Populus euphratica* (Salicaceae). *Canadian Journal of Forest Research*, *30*(3), 482–486. https://doi.org/10.1139/x99-230

Rottenberg, A., & Parker, J. S. (2003). Conservation of the critically endangered Rumex rothschildianus as implied from AFLP diversity. *Biological Conservation*, *114*(2), 299–303. https://doi.org/10.1016/S0006-3207(03)00049-1

Rottenberg, A., Zohary, D., & Nevo, E. (1999). Patterns of Isozyme Diversity and Vegetative Reproduction of Willows in Israel. *International Journal of Plant Sciences*, *160*(3), 561–566. https://doi.org/10.1086/314148

Ruggiero, M. V., Capone, S., Pirozzi, P., Reusch, T. B. H., & Procaccini, G. (2005). Mating System and Clonal Architecture: A Comparative Study in Two Marine Angiosperms. *Evolutionary Ecology*, *19*(5), 487–499. https://doi.org/10.1007/s10682-005-0292-x

Ruggiero, M. V., Reusch, T. B. H., & Procaccini, G. (2005). Local genetic structure in a clonal dioecious angiosperm. *Molecular Ecology*, *14*(4), 957–967. https://doi.org/10.1111/j.1365-294X.2005.02477.x

Sato, T., Isagi, Y., Sakio, H., Osumi, K., & Goto, S. (2006). Effect of gene flow on spatial genetic structure in the riparian canopy tree *Cercidiphyllum japonicum* revealed by microsatellite analysis. *Heredity*, *96*(1), 79–84. https://doi.org/10.1038/sj.hdy.6800748

Segarra‐Moragues, J. G., Palop‐Esteban, M., González‐Candelas, F., & Catalán, P. (2005). On the verge of extinction: genetics of the critically endangered Iberian plant species, *Borderea chouardii* (Dioscoreaceae) and implications for conservation management. *Molecular Ecology*, *14*(4), 969–982. https://doi.org/10.1111/j.1365-294X.2005.02482.x

Sherman-Broyles, S. L., Gibson, J. P., Hamrick, J. L., Bucher, M. A., & Gibson, M. J. (1992). Comparisons of Allozyme Diversity Among Rare and Widespread Rhus Species. *Systematic Botany*, *17*(4), 551. https://doi.org/10.2307/2419726

Sica, M., Gamba, G., Montieri, S., Gaudio, L., & Aceto, S. (2005). ISSR markers show differentiation among Italian populations of *Asparagus acutifolius* L. *BMC Genetics*, *6*(1), 17. https://doi.org/10.1186/1471-2156-6-17

Sun, K., Chen, W., Ma, R., Chen, X., Li, A., & Ge, S. (2006). Genetic Variation in *Hippophae rhamnoides* ssp. *sinensis* (Elaeagnaceae) Revealed by RAPD Markers. *Biochemical Genetics*, *44*(5–6), 186–197. https://doi.org/10.1007/s10528-006-9025-2

Takahashi, D., Isagi, Y., Li, P., Qiu, Y., Setoguchi, H., Suyama, Y., Matsuo, A., Tsunamoto, Y., & Sakaguchi, S. (2022). Stable persistence of relict populations involved evolutionary shifts of reproductive characters in the genus *Tanakaea* (Saxifragaceae). *Journal of Systematics and Evolution*, *60*(6), 1405–1416. https://doi.org/10.1111/jse.12849

Van Tussenbroek, B. I., Valdivia‐Carrillo, T., Rodríguez‐Virgen, I. T., Sanabria‐Alcaraz, S. N. M., Jiménez‐Durán, K., Van Dijk, K. J., & Marquez‐Guzmán, G. J. (2016). Coping with potential bi‐parental inbreeding: limited pollen and seed dispersal and large genets in the dioecious marine angiosperm *Thalassia testudinum*. *Ecology and Evolution*, *6*(15), 5542–5556. https://doi.org/10.1002/ece3.2309

Vega, C., Fernández, V., Gil, L., & Valbuena-Carabaña, M. (2022). Clonal Diversity and Fine-Scale Genetic Structure of a Keystone Species: *Ilex aquifolium*. *Forests*, *13*(9), 1431. https://doi.org/10.3390/f13091431

Viruel, J., Catalán, P., & Segarra-Moragues, J. G. (2014). Latitudinal Environmental Niches and Riverine Barriers Shaped the Phylogeography of the Central Chilean Endemic *Dioscorea humilis* (Dioscoreaceae). *PLoS ONE*, *9*(10), e110029. https://doi.org/10.1371/journal.pone.0110029

Walcott, I., Lanspeary, A., Shams, F., Bredell, P., Cook, E., & Higgisson, W. (2025). On the Precipice of Extinction: Genetic Data in the Conservation Management of In Situ and Ex Situ Collections of the Critically Endangered *Muehlenbeckia tuggeranong* (Tuggeranong Lignum). *Plants*, *14*(12), 1812. https://doi.org/10.3390/plants14121812

Wang, R., Yang, C.-H., Ding, Y.-Y., Tong, X., & Chen, X.-Y. (2018). Weak genetic divergence suggests extensive gene flow at the northeastern range limit of a dioecious Ficus species. *Acta Oecologica*, *90*, 12–17. https://doi.org/10.1016/j.actao.2018.02.002

Zhai, F., Mao, J., Liu, J., Peng, X., Han, L., & Sun, Z. (2016). Male and Female Subpopulations of *Salix viminalis* Present High Genetic Diversity and High Long-Term Migration Rates between Them. *Frontiers in Plant Science*, *7*(MAR2016). https://doi.org/10.3389/fpls.2016.00330

Zhao, J.-L., He, T., & Li, Q.-M. (2010). Contrasting coarse and fine scale genetic structure among isolated relic populations of *Kmeria septentrionalis*. *Genetica*, *138*(9–10), 939–944. https://doi.org/10.1007/s10709-010-9475-7

Zhu, S., Comes, H. P., Tamaki, I., Cao, Y., Sakaguchi, S., Yap, Z., Ding, Y., & Qiu, Y. (2020). Patterns of genotype variation and demographic history in *Lindera glauca* (Lauraceae), an apomict‐containing dioecious forest tree. *Journal of Biogeography*, *47*(9), 2002–2016. https://doi.org/10.1111/jbi.13874

Zorzanelli, J. P. F., Kunz, S. H., Carrijo, T. T., de Miranda, F. D., de Souza, L. C., & da Silva Júnior, A. L. (2022). Bases for genetic conservation of *Freziera atlantica*, an endangered wood species and endemic to the Atlantic Forest hotspot. *Trees*, *36*(3), 1005–1015. https://doi.org/10.1007/s00468-022-02266-4
